# An Innovative Drug Delivery System Loaded with a Modified Combination of Triple Antibiotics for Use in Endodontic Applications

**DOI:** 10.1155/2020/8859566

**Published:** 2020-08-25

**Authors:** Ardavan Parhizkar, Hanieh Nojehdehian, Fahimeh Tabatabaei, Saeed Asgary

**Affiliations:** ^1^Iranian Center for Endodontic Research, Research Institute for Dental Sciences, Shahid Beheshti University of Medical Sciences, Tehran 198396-3113, Iran; ^2^Department of Dental Biomaterials, School of Dentistry, Shahid Beheshti University of Medical Sciences, Tehran 198396-3113, Iran; ^3^Department of Endodontics, Iranian Center for Endodontic Research, Research Institute for Dental Sciences, Shahid Beheshti University of Medical Sciences, Tehran 198396-3113, Iran

## Abstract

The objective of the current study was to introduce “Polylactic co-Glycolic Acid- (PLGA-) Coated Ceramic Microparticles” as an innovative drug delivery system, loaded with a new combination of triple antibiotics (penicillin G, metronidazole, and ciprofloxacin (PMC)) for use in endodontic treatments. Ceramic microparticles were made from *β*-tricalcium phosphate and hydroxyapatite and examined by “Scanning Electronic Microscope (SEM).” Then, fixed amounts of the selected antibiotics were added to a prepared PLGA solution and stirred thoroughly. Next, the prepared ceramic microparticles were dispersed completely in the drugs solution. The deposited “PMC-loaded PLGA-coated ceramic microspheres (PPCMs)” were dried and incubated in phosphate buffer saline (PBS) for 21 days. The drug release from PPCMs was quantified by a UV spectrophotometer. The antimicrobial activity of PPCMs was investigated using the “Agar Plate Diffusion Test (ADT),” “Minimum Inhibitory Concentration (MIC),” and “Minimum Bactericidal Concentration (MBC)” against *Enterococcus faecalis* (*E. faecalis*) and *Aggregatibacter actinomycetemcomitans* (*A.a*). The cell viability test (MTT) was conducted for cytotoxicity against human gingival fibroblasts. SEM micrographs of PPCMs showed spherical-like ceramic microparticles with smooth surfaces. Crystal-like antibiotic particles (chunks) were also found on PPCMs. Initial burst of antibiotics (31 *µ*g/mL, 160 *µ*g/mL, and 18 *µ*g/mL for ciprofloxacin, metronidazole, and penicillin G, respectively, in the first 4 days) followed by gradual and sustained release was observed within a period of 21 days. PPCMs demonstrated pH close to normal physiological environment and antibacterial activity against *E. faecalis* and *A.a* in the first 2 days. MTT showed cell viability of more than 70% for PPCMs after 24 h and 72 h of exposure. In conclusion, PPCMs demonstrated satisfactory release of antibiotics, antibacterial activity against the selected microorganisms, and biocompatibility. Thus, PPCMs may be used to deliver modified triple antibiotics to the root canal system for use in endodontic applications.

## 1. Introduction

Endodontics is a discipline in dentistry, which helps preserve dental structure and maintain its functions in the oral cavity. “Regenerative Endodontics (RE)” has become one of the premier centers of attention in endodontics in recent years [1]. As a result, many dental professionals, clinicians, and specifically, researchers have focused their efforts on regenerative practices. “Endodontic Regeneration Protocol (ERP)” is a novel concept dealing with necrotic open apex teeth. To reach a successful outcome in ERP, achievement of a clean environment through disinfection of the root canal system is an essential need [2].

To promote ERP, employment of intracanal medication for the disinfection of the radicular area is necessary [3]. Calcium hydroxide (CH) has been used as the medicament of choice to create the desired setting in the root canal space for years [4]. In addition to CH, triple antibiotic paste (TAP), which is a combination of ciprofloxacin, metronidazole, and minocycline, has been introduced as an intracanal medication to combat and remove root canal microbiota for ERP [5]. Moreover, it has been shown that modifications of TAP, i.e., double antibiotic paste and modified TAPs, can be applied to fulfil the mentioned purpose. However, there are drawbacks to the use of TAP, including crown discoloration, changes to dentin structure/properties, and its cytotoxic effect in high concentrations [6, 7]. If TAP is to be used for ERP, it should be employed at a concentration of 1–5 mg/mL as recommended/prescribed by the “American Association of Endodontists” [6, 8]. Furthermore, other materials and systems have been considered for ERP to achieve root canal disinfection, namely, calcium hydroxide as an intracanal medicament [8, 9] and the newly devised three-dimensional drug delivery construct, which seems to be a promising potential as a disinfection strategy for RE [10].

Parallel to contemporary schools of thought in medicine, efforts to directly transfer medicaments to the root canal space using different carriers, known as local drug delivery systems (LDDS), have been considered in dentistry [11]. LDDS seem to be responsible for “Target Drug Strategy (TDS).” In TDS, the main objectives are to (a) transfer the maximum/therapeutic concentration of drug(s) directly to the intended tissue/site with decreased adverse effects and (b) release drug(s) in a controlled manner [12]. A variety of drug delivery microvehicles where drugs can be entrapped and taken to the target site have been investigated, e.g., microparticles, nanoparticles, micelles, and liposomes [13, 14].

Studies have shown the importance of hydroxyapatite (HA) as LDDS. HA can mimic the structure of bone, perform as a bone substitute, transfer drugs (e.g., insulin), and act as a vehicle in different situations [15, 16]. *β*-tricalcium phosphate (*β*-TCP) has also been used for drug transfer in bone and bone regeneration as a local drug delivery carrier [17]. Furthermore, other studies have reported the use of biphasic ceramic microparticles, synthesised from HA/TCP as a drug delivery system [18], and ceramic microparticles, as LDDS, in dentistry [19]. Polymers are also considered to transfer drugs to specific targets [20]. Amongst polymers used as a drug delivery system, “Polylactic co-Glycolic Acid (PLGA)” has been considered for different applications due to its excellent degradation properties and ability of drug release [21]. Moreover, PLGA-based drug delivery systems have been used in dentistry to transfer drug(s) to the intended site [22, 23].

The aim of this research was to introduce an innovative local drug delivery system to the root canal area for use in endodontic treatments. This system could transfer the selected antibiotics to the radicular area and release the employed drugs for a period of time. Owing to changes in the combination of drugs, the modified triple antibiotics seemed to be effective on a diverse group of root canal microbiota.

## 2. Methods

### 2.1. Selection of Antibiotics

To select antibiotics for this study, a comprehensive review of intracanal microbiota was conducted, and the dominant microorganisms in the root canal system were studied and listed [24]. For each microorganism, the most commonly used and effective antibiotics were named. Afterward, the effective antibiotics were classified according to their impact on (i) micro‐organisms and (ii) color, and the effective antibiotics with white appearance were chosen. Next, each white-colored antibiotic and its affected microorganisms were listed separately. Then, the effects of the combination of “two white-colored antibiotics” and that of “three white-colored antibiotics” on the dominant microorganisms were distinctly listed and tabled. After careful observation and study of the devised tables, the most efficacious triple antibiotic combination, “penicillin G, metronidazole, and ciprofloxacin (PMC),” was selected for the study.

### 2.2. Synthesis of Ceramic Microparticles

This study was performed using ceramic microparticles. High purity *β*-tricalcium phosphate (*β*-TCP) (Sigma Aldrich Chemicals, USA) and hydroxyapatite (HA) (Sigma Aldrich Chemicals, USA) were used for the preparation of ceramic microparticles. Low-viscosity paraffin oil (Dr. M, Iran) and gelatin (Sigma Aldrich Chemicals, USA) were procured. To make ceramic microparticles, a 6% aqueous solution of gelatin content was initially prepared. 65% of HA and 35% of *β*-TCP were thoroughly mixed on a pad and then filtered using a sieve #60 with an aperture size of 250 *μ*m. The obtained powder was gently added to the gelatin solution and, on a stirrer, was mixed thoroughly at room temperature. The time of stirring and the rpm of the device (2 h/300 rpm) were perfected to form microparticles. The gelatin slurry containing *β*-TCP/HA was then dispersed in paraffin oil in a Petri dish and mixed thoroughly. The excess amount of paraffin was removed using acetone. What remained on the dish were *β*-TCP/HA ceramic microparticles, which were gathered and left in open air at room temperature for 24 hours to dry. Next, they were heated in a ceramic furnace at 550°C for 1 hour (to remove the remaining gelatin) and sintered at 950°C for 1 hour (to strengthen). The microparticles were then completely washed in distilled water to remove the unburned gelatin and afterward, fully dried in an oven [19].

### 2.3. Surface Morphology, Particle Size, and Functional Groups

The surface morphology and diameter of the *β*-TCP/HA ceramic microparticles were observed under “Scanning Electron Microscope (SEM)” (VAT; 5402-GE02-0002/6891, Austria). “Energy Dispersive X-ray Analysis (EDXA)” was conducted to chemically characterize the ceramic microparticles. Oxygen, calcium, and phosphorus were investigated by EDXA and mapped afterward. “Fourier Transform Infrared Spectroscopy (FTIR)” (Bruker, Tensor 27, Germany) was also used to ascertain the functional groups in the microparticles.

### 2.4. Coating of Ceramic Microparticles

PLGA solution (5%) was chosen to coat the ceramic microparticles. To make the solution, 5 grams of the polymer (Sigma Aldrich Chemicals, USA; 50 : 50–504 H) was added to 100 mL of chloroform (Merck, Germany) as the selected solvent. Chloroform was chosen as the solvent since the drugs (antibiotics), to be loaded on the ceramic microparticles, could dissolve in chloroform. PLGA was gently added to chloroform and stirred thoroughly for 30 minutes at room temperature to achieve a homogenous liquid PLGA/chloroform solution. To have the highest possible amounts of the selected antibiotics on ceramic microparticles, their saturation limits (the maximum amount of each antibiotic that could be completely solved in the solvent “chloroform” without being deposited) per milliliter were determined and then added to the PLGA/chloroform solution. 6.2 mg of ciprofloxacin (Alborz Daroo, Iran), 50 mg of metronidazole (Alborz Daroo, Iran), and 12.5 mg of penicillin G (Shifa, Iran) per milliliter were weighed and added separately to the liquid PLGA/chloroform solution. Each amount of the antibiotics was mixed thoroughly for at least 20 min to achieve a homogenous antibiotic/PLGA/chloroform solution and 60 min (minimum) for PMC/PLGA/chloroform solution. Then, 5 mg/mL of the prepared dried ceramic microparticles was added to the PMC/PLGA/chloroform solution and mixed completely. The whole mixture was centrifuged at 4400 rpm for 15 minutes, and the liquid phase was removed. “PMC-loaded PLGA-coated Ceramic Microparticles (PPCMs)” were left at room temperature to dry for 24 hours. Then, PPCMs were frozen-dried using a freeze-drier (Pishgam, Iran) for 48 hours. Obtained PPCMs were further studied with SEM. In a similar manner, equal amount of ceramic microparticles was added to the homogenous liquid PLGA/chloroform solution (without PMC) to be used as the control group (PCM). PCMs were also studied using SEM.

### 2.5. Drug Release

**Figure 1:**
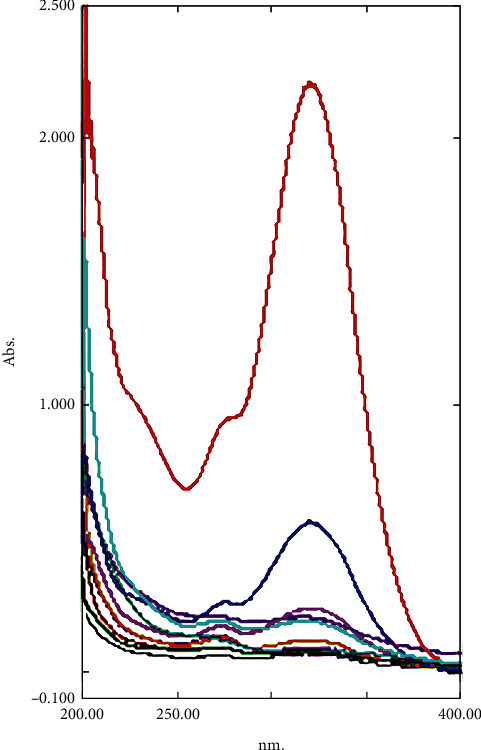
UV spectrophotometry report of the wavelengths specific for each antibiotic, 271 nm for ciprofloxacin, 319 nm for metronidazole, and 200 nm for penicillin G.

To determine the amount of antibiotic release from PPCMs, ultraviolet (UV) spectrophotometry was used. Thirteen milligrams of PPCMs were weighed and added to 3 mL of “Phosphate Buffer Saline (PBS)” with the pH of 7.4 on a six-well plate. The plate was placed in a shaker incubator (Hysc, China) at 70 rpm and 37°C for 21 days. The release extracts were collected every 48 hours and replaced with fresh PBS (3 mL in each well) [25]. The amounts of release were determined using UV spectrophotometry (UV-2501PC/UV-VIS Recording Spectrophotometer, Shimadzu, Japan) at wavelengths specific for each antibiotic, 271 nm for ciprofloxacin, 319 nm for metronidazole, and 200 nm for penicillin G (Figure 1). The remaining amounts of extracts were frozen and sterilized using 0.2 *μ*m syringe filters for antibacterial tests [25]. For determining the short-term release of the selected antibiotics, the release extracts were collected every 30 minutes in a period of 12 hours and then examined by UV spectrophotometry.

### 2.6. Encapsulation Efficacy

Thirteen milligrams of PPCMs were dissolved in 5% (w/v) sodium dodecyl sulfate in 0.1 M NaOH and stirred overnight. Then, the solution was centrifuged at 4400 rpm for 15 minutes, and the liquid phase (extract) was obtained. Next, the extract was read using UV spectrophotometry. In addition, the UV absorbance against PCMs in the related wavelengths was measured. To determine the “Encapsulation Efficacy,” the following equation was used [26]:

(1) $$\begin{array}{c} \text{Encapsulation Efficiency }=\left(\frac{\text{actual}}{\text{theoretical drug loading}}\right)\times 100\text{\%.} \end{array}$$

### 2.7. pH

To determine pH, a digital pH meter (Jenway, England) was used. Thirteen milligrams of PPCMs were weighed and added to 3 mL of PBS with the pH of 7.4 on a six-well plate. Then, the plate was placed in a shaker incubator (Hysc, China) at 70 rpm and 37°C for 7 days. The release extracts were collected every 24 hours and replaced with fresh PBS (3 mL in each well). pH was read using a digital pH meter.

### 2.8. Antibacterial Tests

To evaluate the antimicrobial activity of PPCMs, “Agar Diffusion Test (ADT)” and “Minimum Inhibitory Concentration (MIC)/Minimum Bactericidal Concentration (MBC) tests” (Colorimetric Broth Microdilution Method) on two microorganisms, *Enterococcus faecalis* (*E. faecalis*) and *Aggregatibacter actinomycetemcomitans* (*A.a*), were performed. *E. faecalis* is a Gram-positive microorganism, considered as one of the most dominant microorganisms in endodontic lesions and found in necrotic open apex teeth. *A.a* is a Gram-negative microorganism and can be found in the radicular space; however, *A.a* shows completely different characteristics in comparison with *E. faecalis*.

To perform ADT, “Brain Heart Infusion (BHI)” agar (Merck, Germany) plates were prepared. Since two microorganisms were tested, two jars had to be used, each with their respective bacterium. The jar containing *E. faecalis* was incubated for 24 h at 37°C, whereas the jar containing *A.a* was incubated for 72 h at 37°C. From each microorganism colony, a 0.5 McFarland suspension was prepared. Then, each suspension was cultured on the corresponding agar plate. Next, on each plate, six wells were created.

The antibiotic-release extracts of PPCMs were collected every 24 hours for 7 days. 50 *μ*L of each extract was taken and sterilized using a 0.2 *μ*m syringe filter. The sterilized extracts were then transferred to the defined created well (well 1). Two other wells contained (a) the free form of the three employed antibiotics that were not loaded on the microparticles and had a concentration exactly the same as the maximum concentrations of the released drugs from PPCMs and (b) PCMs, which were considered as the control group. Two other wells containing PBS and water were regarded as negative control groups, whilst the remaining well with chlorhexidine 0.2% was considered as the positive control group, affecting the bacteria directly. For each microorganism, 3 plates were used. The prepared plates were placed in a jar and transferred to an incubator (Memmert, Germany). After 24 and 72 hours of incubation for *E. faecalis* and *A.a*, respectively, the diameters of growth inhibition zones around the wells were measured in millimeters using a standard ruler.

To perform MIC/MBC tests, 100 *µ*L of sterile BHI was added to each of the designated wells on a 96-well plate (SPL, Korea). Then, 100 *µ*L of the prepared free form of the combination of the three chosen antibiotics, that was not loaded on the microparticles and had a concentration exactly the same as the maximum concentrations of the released drugs from PPCMs, was added to the first well of the designated row and diluted in the next wells (1 : 4, 1 : 6, 1 : 8, and so forth). This was done in three rows. Then, 0.5 McFarland suspension from *E. faecalis* was added to the medium in all designated wells except for the negative control group. The positive control group included medium and bacteria, and the negative control group only had the medium. After 24 h of incubation, 10 *µ*L resazurin (0.01%) was added to the tested wells. Then, the plate was incubated for 2 hours. After incubation, the change in color was observed. The well, which was the most transparent, was considered as MIC. Same procedure was conducted for *A.a*; however, for the first incubation period, *A.a* was incubated for 72 hours. From the concentrations leading to MIC, small amounts of the prepared free form of the antibiotic solution were transferred to prepared agar plates. The corresponding microorganism from 0.5 McFarland suspension was added to the plates, and then, they were incubated at 37°C for 24 h. The minimum concentration, which showed no bacterial growth, was considered as MBC.

### 2.9. Assessment of Cytotoxicity

The cytotoxicity of microparticles was evaluated using an indirect method (extract method) on human gingival fibroblasts (HGF1-PI1, NCBI code: C165, Pasture Institute Cell Bank, Iran) according to the biological evaluation of medical devices-Part 5: tests for in vitro cytotoxicity (ISO 10993-Part 5, 2009).

The drug-loaded (PPCMs) and unloaded (PCMs) microparticles (4.5 mg/mL) were incubated in “Dulbecco's Modified Eagle's Powder Medium (DMEM)” (Gibco, UK) for 24 h (37°C and 70 rpm) in order to obtain the extract of microparticles (ISO10993-12, 2012). Then, the extracted solutions of microparticles were sterilized using a 0.2 *μ*m sterile syringe filter.

The HGF cells were seeded at logarithmic growth phase with the density of 2,500 cells per well (10 wells for each sample, *n* = 10) of 96-well culture plates (SPL, Korea) in DMEM supplemented with 1% antibiotic (penicillin and streptomycin) and 10% fetal bovine serum (FBS) (Gibco, UK). After 24 h of incubation at 37°C and 5% CO_2_ (cell confluency∼50%), the medium was removed and replaced with 100 *µ*L of the extracted solutions of microparticles (supplemented with 10% FBS without antibiotic). The cells treated with regular culture medium only (supplemented with 10% FBS without antibiotic) and water only were considered as the negative (no cytotoxicity and 100% viability) and positive (severe cytotoxicity and <10% viability) control groups for cell viability, respectively. Also, the cells treated with DMEM (supplemented with 10% FBS without antibiotic) containing free forms of three antibiotics (unloaded on microparticles) at a concentration exactly the same as the maximum concentrations of drugs released from the microparticles (31 *µ*g/mL, 160 *µ*g/mL, and 18 *µ*g/mL for ciprofloxacin, metronidazole, and penicillin G, respectively) were considered as the control group for antibiotic-loaded microparticles.

At 24 h and 72 h after treatment, the medium in each well was removed and replaced with the culture medium containing 10% MTT (methyl thiazolyl tetrazolium) dye (5 mg/mL stock solution; Sigma-Aldrich Chemicals, Germany). After 3 h of incubation at 37°C, the MTT medium was completely removed and replaced with the same volume of dimethyl sulfoxide solvent (DMSO; Sigma-Aldrich Chemicals, Germany) to dissolve purple formazan crystals. The optical density (OD) was read at 570 nm (main) and 620 nm (reference) wavelengths by an Elisa Reader (Anthos 2020, Austria). The percentage of cell viability (% of viable cells) was calculated using the following formula:

(2) $$\begin{array}{c} \text{cell viability}\left(\text{percentage of control}\right)=\left[\frac{\text{OD of treated sample}}{\text{OD of untreated sample}\left(\text{control cells}\right)}\right]\times 100. \end{array}$$

### 2.10. Statistical Analysis

The results were analyzed using GRAPHPAD PRISMV.6.07 software (GraphPad Software, USA) and one-way ANOVA, followed by Tukey's post hoc test, and the relevant graph was plotted. A value of *P* < 0.05 indicated statistically significant differences either with the negative control group or between the experimental groups.

## 3. Results

### 3.1. Selection of Antibiotics

In this research, PMC was introduced/used as a new combination of triple antibiotics due to its wide antimicrobial scope and impact against microorganisms.

### 3.2. Characterization of Microparticles

#### 3.2.1. Scanning Electron Microscopy (SEM)

**Figure 2:**
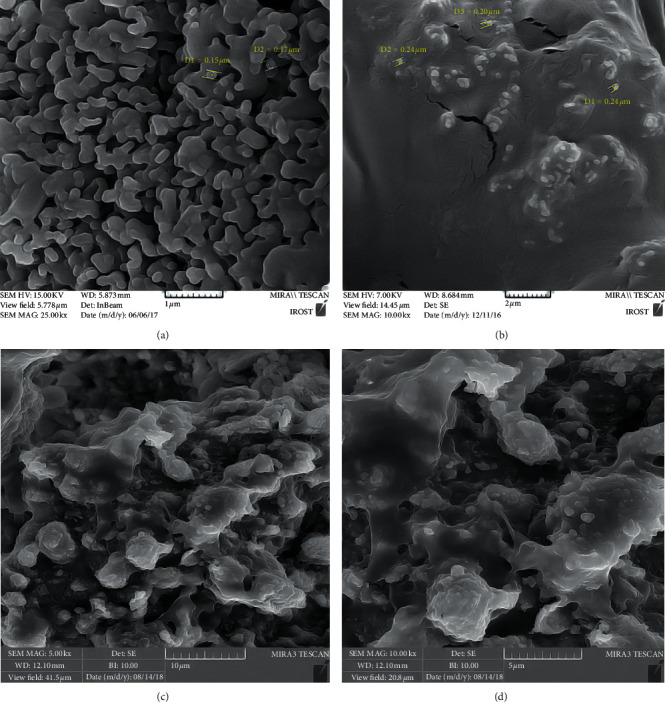
Scanning electron microscope micrographs of ceramic microparticles (a) before being coated, (b) after being coated with PLGA, and (c), and (d) loaded with the new combination of antibiotics–with magnification of 5x and 10x.

The morphology and size of the ceramic microparticles were observed and determined by SEM. As shown in Figure 2(a), ceramic microparticles had smooth surface with nearly spherical shape and were 0.15 *µ*m–0.17 *µ*m large. When coated with PLGA, the microparticles were found to be more separate while covered by the polymer, and an increase in size, i.e., 0.20 *µ*m–0.24 *µ*m, was observed (Figure 2(b)). When loaded with PMC, they were found to have chunks of antibiotics (Figure 2(c)). However, as time passed, drug loss could be seen (Figure 2(d)).

#### 3.2.2. Energy Dispersive X-Ray Analysis (EDXA)

**Figure 3:**
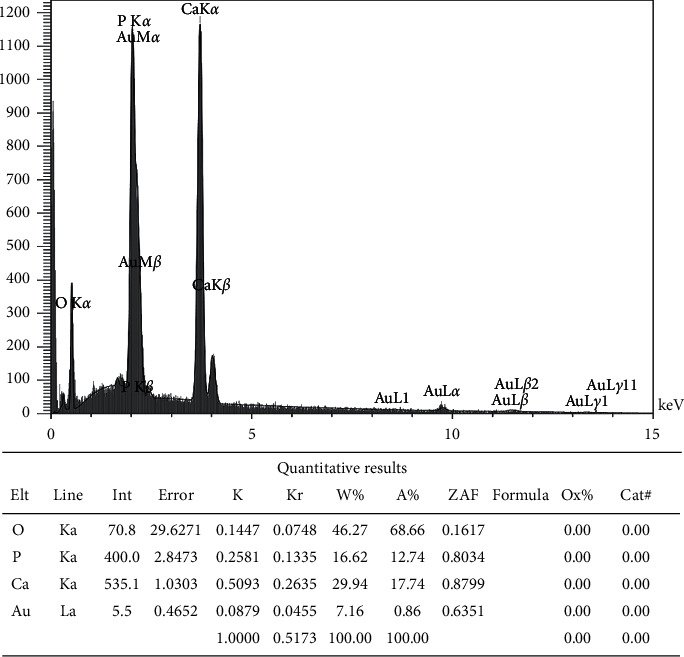
EDXA of the prepared ceramic microparticles. EDXA showed that the weight percentage of calcium was more than phosphate, and the Ca/P ratio (w %) in the ceramic microparticles was 1.8.

EDXA was used to determine the weight percentages of calcium, phosphate, and oxygen. The obtained results showed that the weight percentage of calcium was more than that of phosphate (29.94%–16.92%). Furthermore, the Ca/P ratio in the ceramic microparticles (without PMC and polymeric coating) was 1.8 (or 1.39 in molarity) (Figure 3).

#### 3.2.3. Fourier-Transform Infrared Spectroscopy (FTIR)

**Figure 4:**
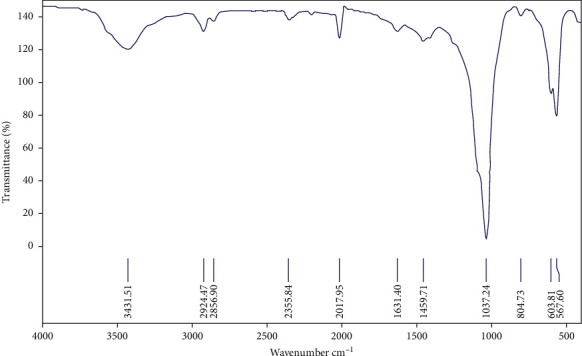
FTIR of ceramic microparticles. The FTIR showed a peak vibration at wavenumbers 1490 cm^−1^/1640 cm^−1^/3431.51 cm^−1^ and 1037.24 cm^−1^, which corresponded to OH-1 and PO4-3 stretches of HA/*β*-TCP, respectively.

The spectral interpretation of FTIR showed peak vibrations near wavenumbers 1490 cm^−1^/1640 cm^−1^/3431.51 cm^−1^, which contributed to OH^−1^ stretches. In addition, a peak vibration was seen at wavenumber 1037.24 cm^−1^, which corresponded to PO_4_^−3^ stretches. These findings resembled the existence of HA and *β*-TCP in the prepared ceramic microparticles (Figure 4).

### 3.3. pH

The result of our study showed that the extracts of PPCMs demonstrated a pH close to the normal physiological environment over 7 days.

### 3.4. Drug Release

**Figure 5:**
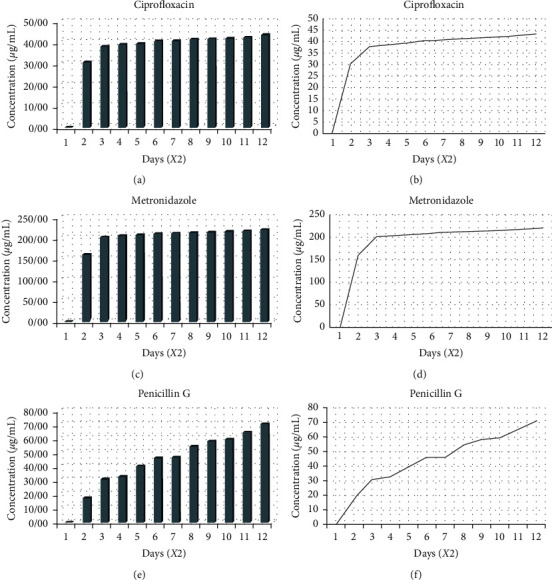
The in vitro drug release profile of the three antibiotics from PPCMs over 21 days. The cumulative drug-release concentration of the antibiotics in “Phosphate Buffered Saline (PBS)” is expressed in *µ*g/mL. As it can be seen in the profile, ciprofloxacin and metronidazole had a burst release in the first 4 days but reached a relative plateau on the other days. However, penicillin G continued its sustained release throughout the 21 days of the experiment (*Note.* In the above profiles, “Days” should be multiplied by two, as indicated in the horizontal axis).

Figure 5 depicts the profile of drug release over a period of 21 days. The antibiotics were released from PPCMs over three weeks, with a burst release on the first four days. On day 4, the maximum amounts of release were 31 *μ*g/mL, 160 *μ*g/mL, and 18 *μ*g/mL for ciprofloxacin, metronidazole, and penicillin G, respectively. After the first 4 days, there was almost a plateau in the release patterns of ciprofloxacin and metronidazole, whilst penicillin G continued its gradual release on the other days.

### 3.5. Antibacterial Activity

ADT exhibited that the extracts collected from PPCMs presented antibacterial activity against *E. faecalis* and *A.a* in the first 48 h. The extracts showed growth inhibition zones of 25.3 mm ± 0.4 after 24 h and 13 mm ± 0 after 48 h against *E. faecalis* and 42.3 mm ± 2.0 after 24 h and 30 mm ± 0 after 48 h against *A.a*. However, there was no growth inhibition zone on day 3 for the microorganisms. The extracts collected from PCMs (i.e., polymer/the microparticle control group) showed no antibacterial effect on the first 3 days and onward. MIC and MBC tests showed that the chosen combination of antibiotics at the determined concentrations had the antibacterial effect against *E. faecalis* and *A.a.* The MIC test showed that at the concentration of 1 : 32 and 1 : 256 of the maximum concentrations of the released antibiotics from PPCMs, PMC could prohibit the bacterial growth of *E. faecalis* and *A.a*, respectively. The MBC test showed similar results for the antibiotics, 1 : 32 and 1 : 128 for *E. faecalis* and *A.a*, respectively (Table 1). Based on our results, PPCMs could be used for immediate antibacterial activity over a period of 2 days.

MIC, minimum inhibitory concentration; MBC, minimum bactericidal concentration; ADT, agar diffusion test.

### 3.6. Cytotoxicity

According to ISO-10993-5 (2009) standard (in vitro cytotoxicity testing), the percentage of viability of untreated control cells (exposed to regular culture medium alone, without microparticle extracts or free antibiotics) is considered 100%, and a material decreasing the percentage of cell viability by more than 30% compared with the untreated control group is considered cytotoxic (viability < 70%).

**Figure 6:**
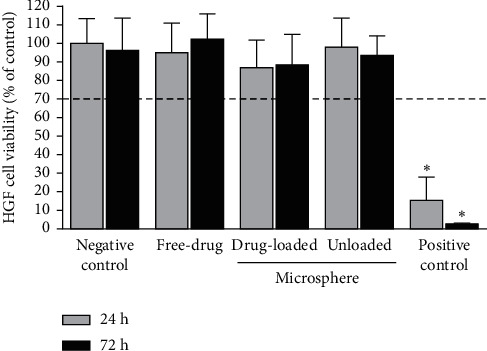
Cell viability percentage of treated HGF cells compared to untreated HGF cells (negative control; no cytotoxicity; 100% viability). Positive control: water-treated HGF cells (severe cytotoxicity).

As shown in Figure 6, 24 h and 72 h after exposure, all of the test groups (free drug, unloaded microparticles, and drug-loaded microparticles) showed a viability similar to that of the untreated negative control group (∼100%). Generally, viability of HGF cells treated with three test groups (at 24 h and 72 h after exposure) was not significantly different (*P* > 0.05) from each other and from that of the negative control group. According to the results, significant time-dependent cytotoxicity in HGFs was noted only in the positive control group (viability <15%).

## 4. Discussion

In the present research, polymer-coated ceramic microparticles were devised as an innovative drug delivery system; it showed to be bioactive, biocompatible, and capable of releasing loaded antibiotics. Furthermore, the current investigation introduced a new combination of triple antibiotics for use in endodontic treatments. The antibacterial combination had white appearance and could affect intracanal microbiota.

The used ceramic microparticles in this study were “150 nm–170 nm” and “200 nm–240 nm” large before and after being coated with PLGA, respectively, which was in accordance with the defined range of an ideal drug delivery system (2 nm–1 *µ*m) [27]. In addition, SEM micrographs of the ceramic microparticles showed spherical-like vehicles with smooth surface, which could result in less inflammatory response in the target sites/tissues. This outcome was similar to that of a study by Victor who expressed the same result in his research [19]. The prepared ceramic microparticles were made from *β*-TCP/HA as the core of our drug delivery system. *β*-TCP/HA has been considered as a scaffold for the formation of tissue-engineered bone [28]. *β*-TCP is biocompatible/biodegradable/bioactive, and HA is able to prepare a matrix for bone formation [29]. Thus, if the synthesised ceramic microparticles were placed next to the apical foramen and in close contact with the bone, hard tissue induction would also be expected. However, further research is needed to favor this hypothesis. Poly lactic co-glycolic acid (PLGA) was the polymer of choice as the coating material in our study. PLGA is a member of the polyester family, and its nontoxicity, processability, biodegradability, and having good biodegradation rate have gained attention in drug delivery systems [30].

In the current research, a comprehensive intracanal microbiological/antimicrobial study was conducted to define and determine effective intracanal medication. Various types of microorganisms are found in infectious root canal systems [31]; therefore, a purpose of endodontic treatment is to remove as many bacteria as possible using different methods, e.g., aseptic treatment protocols, chemomechanical preparation, and so forth. Local use of antimicrobial medication has been considered as an adjunct to systemic and conventional antibiotics. On the other hand, due to the complexities and nature of microorganisms found in the radicular system, it is highly unlikely to combat root canal microbiota with one single antibiotic. Since both facultative and obligate aerobes and anaerobes can be found in the radicular area, a combination of antibiotics should be considered for root canal disinfection [24].

PMC, in comparison with other studied combinations of antibiotics, showed to (a) be more effective and (b) have a wider scope over the dominant microorganisms in the root canal system. The results of the present study indicated that the combination of penicillin G, metronidazole, and ciprofloxacin could affect most important intracanal microorganisms when compared to other studied combinations of triple antibiotics, including TAP. In the introduced triple combination, penicillin G replaced minocycline in TAP whilst metronidazole and ciprofloxacin were found in both combinations. Penicillin G is effective against a more diverse group of intracanal microorganisms compared to minocycline. This dominance includes *E. faecalis*, an important microorganism in endodontic failure. *E. faecalis* and *A.a* are both sensitive to ciprofloxacin and penicillin G but are resistant against metronidazole. Our modification of the triple antibiotics was in line with other research studies where minocycline was replaced/removed. Thomson et al. used amoxicillin; nevertheless, amoxicillin caused delayed discoloration in the treated teeth [32]. Using minocycline-removed paste (double antibiotic paste) has also been reported in several studies; however, tooth discoloration is still a challenge [6, 33]. Penicillin G has a white appearance; nonetheless, further research is necessary to investigate if PMC does not change the color of the tooth structure. Moreover, the present study demonstrated that the combination of PMC could affect more microorganisms that each antibiotic alone, which conformed to a study by Chuensombat [34]. Therefore, this research introduces PMC as a new combination of triple antibiotics for possible use in endodontic applications and against root canal microorganisms.

Burst release of PMC could be seen in the first 4 days, with ciprofloxacin, metronidazole, and penicillin G at the release concentrations of 31 *μ*g/mL, 160 *μ*g/mL, and 18 *μ*g/mL, respectively. The burst release, owing to the solubility of antibiotics in water and increase of hydrophilicity, was followed by the gradual and then sustained release of the antibiotics, which could also be related to the gradual degradation of the PLGA coating on the microparticles. Release of antibiotics from PLGA seems to be related to the interaction between the diffusion and degradation processes in the polymer and is primarily based upon the mass degradation of PLGA [35]. The burst release is also similar to dosing regimen of antibiotics where double dose of the drug is initially prescribed [36]. The rates of release of three antibiotics were different from one another, ciprofloxacin being the slowest. This could be related to different chemistry of antibiotics and the type of bond each antibiotic had with the coating polymer.

ADT inhibition zones demonstrated that the extracts collected from PPCMs showed severe antibacterial activity against *E. faecalis* and *A.a* in the first two days and thus could be able to eliminate different types of microorganisms from the root canal system in a short period of time. The disappearance of the inhibition zone on the third day may be related to the lower concentrations of antibiotics that were not sufficient to diffuse in agar in order to reveal their antibacterial effect. The latter finding is similar to the results of some other studies, which expressed doubts about the results of ADT [37, 38]. Furthermore, MIC and MBC tests showed that lower dilutions of the new combination of PMC could inhibit the growth of the chosen microorganisms (Table 1).

PBS was chosen as the release medium for the extracts in the study. PBS was also used by Torshabi and Tiwari for the same procedure since it could resemble human body conditions. In another study, distilled water was used as the drug-release medium [39]. In our research, water was used for the preparation of the free form of antibiotics (control group).

In our current study, we examined the cytotoxicity of PPCMs on “Human Gingival Fibroblast (HGF)” cells. Our research showed that PPCMs and PCMs demonstrated excellent biocompatibility toward the mentioned cells (viability >70%) whilst water (positive control group) was cytotoxic on HGF cells (viability <30%). In addition, the viability of HGF cells affected by PPCMs was not significantly different from that of PCMs. In addition, the effect of PPCMs on HGF cells was close to that of the free form of antibiotics. Therefore, PPCMs could be considered safe to be used in the radicular system.

A limitation to our study was that the 7+ day contact between the microparticles and HGF cells should be evaluated since we examined 24 h and 72 h evaluations. Furthermore, prior to use in clinical setting, the efficacy and biocompatibility of PPCMs must be tested in animal models. The inflammatory response in the animal models, due to the degradation products of the polymer and microparticles, should be properly investigated. Moreover, PPCMs should be transferred to the root canal system via a suitable vehicle/carrier, for instance water-based solutions and/or intracanal cements, which are not supposed to have adverse effects on the PPCMs and its components. Nevertheless, exploration of suitable vehicles/carriers requires further research.

## 5. Conclusions

The idea of the current study follows the present day concepts of transferring medicaments locally to the root canal system. The innovative PPCMs can be used to deliver antimicrobials to the radicular area. Considering the outcomes of our study, it was concluded that the release pattern of the triple antibiotics from PPCMs was feasible. The PPCMs expressed neutral pH, demonstrated sufficient release, showed acceptable antibacterial activity against *E. faecalis/A.a*, and exhibited cell biocompatibility. Therefore, PPCMs can be regarded as an innovative mean of transferring antibiotics to the root canal area and provide localized and sustained release of drugs. This innovative drug delivery system can open a promising new prospective concerning the current aspects of drug delivery to the radicular space and for regenerative endodontics.

## Figures and Tables

**Table 1:** Antibacterial activity: the results of the released PMC from PPCMs on “*Enterococcus faecalis* (*E .faecalis*)” and “*Aggregatibacter actinomycetemcomitans* (*A.a*).”

Microorganism	MIC	MBC	ADT inhibition zone (mm) (mean ± SD)		
			24 h	48 h	72 h
*Enterococcus faecalis* (*E. faecalis*)	1 : 32	1 : 32	25.3 ± 0.4	13 ± 0	0
Aggregatibacter actinomycetemcomitans (*A.a*)	1 : 256	1 : 128	42.3 ± 2.0	30 ± 0	0

## Data Availability

The data used to support the findings of this study areavailable from the corresponding author upon request.
